# A primary care, electronic health record-based strategy to promote safe drug use: study protocol for a randomized controlled trial

**DOI:** 10.1186/s13063-014-0524-x

**Published:** 2015-01-27

**Authors:** Kamila Przytula, Stacy Cooper Bailey, William L Galanter, Bruce L Lambert, Neeha Shrestha, Carolyn Dickens, Suzanne Falck, Michael S Wolf

**Affiliations:** Division of General Internal Medicine, Feinberg School of Medicine at Northwestern University, 750 N Lake Shore Drive, 10th Floor, Chicago, IL 60611 USA; Division of Pharmaceutical Outcomes and Policy at the UNC Eshelman School of Pharmacy, Chapel Hill, NC USA; Medicine and Pharmacy Practice at the University of Illinois at Chicago, Chicago, IL USA; Northwestern Center for Education and Research on Therapeutics (CERT), Chicago, IL USA; Department of Communication Studies, Center for Communication and Health at Northwestern University, Chicago, IL USA; College of Nursing at the University of Illinois in Chicago, Chicago, IL USA

**Keywords:** Electronic health record, Health literacy, Prescription medication

## Abstract

**Background:**

The Northwestern University Center for Education and Research on Therapeutics (CERT), funded by the Agency for Healthcare Research and Quality, is one of seven such centers in the USA. The thematic focus of the Northwestern CERT is ‘Tools for Optimizing Medication Safety.’ Ensuring drug safety is essential, as many adults struggle to take medications, with estimates indicating that only half of adults take drugs as prescribed. This report describes the methods and rationale for one innovative project within the CERT: the ‘Primary Care, Electronic Health Record-Based Strategy to Promote Safe and Appropriate Drug Use’.

**Methods/Design:**

The overall objective of this 5-year study is to evaluate a health literacy-informed, electronic health record-based strategy for promoting safe and effective prescription medication use in a primary care setting. A total of 600 English and Spanish-speaking patients with diabetes will be consecutively recruited to participate in the study. Patients will be randomized to receive either usual care or the intervention; those in the intervention arm will receive a set of print materials designed to support medication use and prompt provider counseling and medication reconciliation. Participants will be interviewed in person after their index clinic visit and again one month later. Process outcomes related to intervention delivery will be recorded. A medical chart review will be performed at 6 months. Patient outcome measures include medication understanding, adherence and clinical measures (hemoglobin A_1_c, blood pressure, and cholesterol; exploratory outcomes only).

**Discussion:**

Through this study, we will be able to examine the impact of a health literacy-informed, electronic health record-based strategy on medication understanding and adherence among diabetic primary care patients. The measurement of process outcomes will help inform how the strategy might ultimately be refined and disseminated to other sites. Strategies such as these are needed to address the multifaceted challenges related to medication self-management among patients with chronic conditions.

**Trial registration:**

Clinicaltrials.gov NCT01669473.

## Background

According to the Institute of Medicine, more than 1.5 million preventable drug events occur each year in the USA [1]. A third of these occur in outpatient settings, at a cost of approximately $4.2 billion annually [1]. Ensuring safe medication use is likely to become increasingly important as the number of adults taking prescription medications has risen dramatically [2]. Nearly half of Americans take at least one prescription medication, and one in ten takes five or more drugs, an increase of 70% since 1999 [2].

Despite frequent use, many adults struggle to take medications, with estimates indicating that only half of adults take drugs as prescribed [3]. Medication use may be particularly challenging for diabetic patients, who must often manage multiple comorbidities and complex multidrug regimens. Patients with low health literacy and limited English proficiency are likely to experience even greater confusion and negative medication-related outcomes, emphasizing the need for innovative and effective strategies to promote positive medication-related outcomes in this vulnerable population [4-6].

To address these challenges, we designed a health literacy-informed, electronic health record based strategy for promoting safe and effective prescription medication use among English and Spanish-speaking patients with diabetes mellitus. This project is one of four thematically related projects within the Northwestern University Center for Education and Research on Therapeutics (CERT). Funded by the Agency for Healthcare Research and Quality, the ‘Tools for Optimizing Medication Safety’ CERT is one of seven such centers created to promote optimal use of medications and to raise patient awareness of drug risks and benefits. In this paper, we provide an overview of our intervention, summarize evaluation activities, and discuss the sustainability and potential dissemination of our novel strategy.

## Methods/Design

This study seeks to reduce the patient confusion that often leads to unintentional medication non-adherence or medication errors. To promote sustainability and dissemination, we are utilizing an electronic health record platform to automate the delivery of patient-centered medication information materials. The specific aims of the study are as follows: (1) to refine and field test an electronic health record strategy for generating and distributing low-literacy prescription information for English- and Spanish-speaking primary care patients with diabetes; (2) to assess the process of the electronic health record intervention and its fidelity for providing understandable and actionable prescription information for patients at the point of prescribing; and (3) to evaluate the effectiveness of the electronic health record-based strategy to improve medication understanding, reconciliation, regimen consolidation, and adherence, compared with standard care. We have an additional exploratory aim to examine the effect of the intervention on surrogate clinical outcomes; hemoglobin A_1_c, low-density lipoprotein cholesterol and blood pressure.

This study is being conducted at the University of Illinois Hospital and Health Sciences System, an urban, academic medical center located in Chicago, which uses Cerner Powerchart®, a common electronic health record system. Patients are enrolled from three primary care sites: a resident-staffed general medicine clinic, a medicine/pediatrics clinic, and a faculty primary care clinic. These clinics serve a diverse, low-income patient population that is approximately 65% African American and 20% Hispanic. An estimated 36% of general medicine clinic patients have limited health literacy. Adjusted for patient volume, approximately 25% of these low literacy patients have been diagnosed with diabetes. The current standard of care in this clinic is for patients to receive a basic after-visit summary that describes the medical visit and includes a list of medications prescribed to the patient according to the electronic health record. This summary has not been designed according to health literacy best practices and patient education materials are not routinely given as part of standard care.

We will recruit 600 patients from participating clinics in no set proportion and randomize each to receive either standard care or the intervention. Participants will be eligible if they: (1) are over 18 years old; (2) have a documented diagnosis of diabetes; (3) are currently taking at least three daily, chronic, non-PRN (not pro re nata) medications; (4) speak English or Spanish; (5) have no intention to change clinics within the next year; (6) score 4 or higher on a six-item cognitive screener [7]; (7) are, by self-report, primarily responsible for taking their own medications; and (8) were prescribed a new medication, or have a dose, refill, or frequency change of a current medication for hypertension, hyperlipidemia, or diabetes mellitus during their index clinic visit.

Potentially eligible patients (criteria 1 to 4 above) are first identified via a review of their electronic health records. A list of those patients with a clinic appointment scheduled within the next three months is then compiled and sent to clinic physicians via secure email. Physicians have the opportunity to indicate which potentially eligible patients should not be contacted, owing to severe illness or cognitive concerns. With physician approval, patients will be approached by the research assistant to confirm eligibility following their doctor’s appointment. Research assistants will speak with potential participants after receiving an electronic message that they have been prescribed a new medication or received a changed dose or frequency of a medication of interest. Eligibility criteria will be confirmed before patients provide written consent and are enrolled into the study. The study has been approved by institutional review boards at the University of Illinois in Chicago (2012–0262) and Northwestern (STU00064056) and is also registered on clinicaltrials.gov (NCT01669473).

Patients in the intervention arm will receive three electronic health record-generated items at their clinic visit: (1) a pre-visit medication list, (2) medication information sheets, and (3) a post-visit medication list. These materials were designed by study team members and incorporate health literacy best practices, with Lexile analyses confirming a readability level of <8th grade [8,9]. The medication sheets and pre-visit medication list were previously tested in a National Institutes of Health study (R18HS17220). All materials were translated into Spanish using a committee-based approach and pilot tested among English- and Spanish-speaking patients (*N* = 9) to ensure comprehensibility [10]. The materials are as follows:

1. The pre-visit medication list is generated on check-in and includes all medications prescribed to the patient according to their electronic health record. It lists the dosage, frequency and indication for all medications. The instructions on the form ask patients to report how much of each medication they take on a daily basis and if they have any concerns (need refill, cost, and so on) for any of the drugs. Patients are then asked to add any additional prescription or over-the-counter medications they are using and to remove medications that they are no longer taking. Forms are designed to be shared with providers during the clinic visit to promote medication reconciliation.
2. If the patient is prescribed a new or dose-adjusted medication for diabetes, hyperlipidemia, or hypertension, a medication information sheet for that medicine will print automatically in the patients’ preferred language (English or Spanish). The medication information sheet is a one-page, plain-language information sheet that describes the medication’s indication for use, general directions, and side effects. It is designed to be patient-friendly and to provide essential need-to-know information for each drug.
3. Following the visit, patients receive an updated post-visit medication list, which reflects any changes made to the pre-visit medication list as a result of their visit that day. The post-visit medication list includes all of the patient’s medications, listing dosage, indication, and the times each medication should be taken. It is intended to help patients organize their regimen according to the Universal Medication Schedule [11]. The Universal Medication Schedule ‘grounds’ medication use by providing more explicit times to describe when medicine should be taken (morning, noon, evening, bedtime) [5]. This concept was reviewed and recommended by the Institute of Medicine, the US Pharmacopeia, and the National Council for Prescription Drug Programs as a health literacy best practice. Patients in the intervention arm will receive this post-visit medication list, while those in usual care will receive a standard ‘after-visit summary’ recently implemented at the University of Illinois Hospital and Health Sciences System to comply with meaningful use requirements [12]. This standard after-visit summary is the only educational print material that patients in the usual care arm routinely receive after their clinic visit.

We will interview each patient in person at baseline, immediately following the index clinic visit, and one month after the index visit. At 6 months, a chart abstraction will be conducted to extract clinical surrogate outcome data (hemoglobin A_1_c, blood pressure, and low-density lipoprotein cholesterol).

### Process outcomes

As part of the intervention delivery, we will explore process outcome measures, such as the proportion of patients who automatically received the pre-visit medication list, the proportion of patients who used the intervention materials at home, and whether the intervention materials prompted physician-patient discussion.

### Study outcomes

This study is designed to target four key medication-related outcomes: medication reconciliation, medication understanding, regimen consolidation, and medication adherence.

#### Medication reconciliation

Numerous studies have shown discrepancies between the medications that patients report taking and those listed in their medical chart, undermining the quality and safety of clinical care [13-16]. The pre-visit medication list is designed to prompt greater physician-patient communication about medication use and to ensure that self-reported medication use matches the medications listed in the patient’s chart. To measure medication reconciliation, we will ask patients, during the one-month follow-up interview, what medications they are taking. We will compare self-reports with the medications listed in their chart. Research assistants will note any discrepancies and ask patients whether they are taking any medications that were not named but are listed in their charts. Similarly, the research assistant will inquire after any medications that the patient reports taking but are not on their medication list. This outcome will then be coded as: (1) no discrepancies present between self-report and medical chart or (2) discrepancies present. The number of and type of discrepancies will also be recorded and forwarded to supervising physicians in the research team for review, with clinically significant discrepancies being communicated to the patient’s primary care doctor immediately. This coding schema is consistent with previous studies on medication reconciliation [14,15].

#### Medication understanding

Numerous studies have found limited literacy skills and limited English proficiency to be associated with poorer recall of medication names and indications, inadequate understanding of prescription instructions and precautions, and poorly demonstrated use of medications and devices [11,17]. To measure patients’ understanding of their medication regimens, research assistants will guide each patient through a pill-dosing demonstration. Specifically, patients will be given a tray with 24 compartments, each representing one hour of the day. Patients will be oriented to the tray and then asked to show how they take each of their medications over a 24 hour period of time, using the tray and colored beads that represent pills. Research assistants will record when patients indicate that they take their medications, including dose, frequency, and spacing between doses; this will be coded as correct or incorrect, based on pre-established coding criteria [18].

#### Regimen consolidation

As part of this dosing exercise, we will also measure patients’ consolidation of their medication regimen. Research indicates an inverse relationship between dosing frequency and adherence [19-21]. Despite this, a recent study by our team demonstrated that many patients unnecessarily overcomplicate medication use by not consolidating a complex regimen so that it can be taken the minimum number of times per day [5]. Using the data from the dosing exercise, we will measure regimen consolidation, defined as the number of times per day when medication is taken, as indicated in the dosing exercise.

#### Medication adherence

Non-adherence to prescribed medications is well documented, highly prevalent, and common among patients with chronic conditions, with nearly half of patients taking less than 80% of their prescribed doses [3,22-24]. For this study, where data on adherence to multiple medications is collected, using technology such as medication event monitoring systems is not feasible. We are therefore following other adherence measurement guidelines and triangulating multiple measures of adherence [25]. Medication adherence will be assessed using the Morisky general measure of adherence, a directly observed pill count, and a 4-day self-reported assessment of pills missed [26].

### Key covariates

We will collect data on a number of key covariates, including age, sex, English proficiency, comorbidities, medication and healthcare utilization, income, and race and ethnicity. Health literacy will be measured using the Newest Vital Sign [27].

### Analyses

A sample size of 600 patients, 300 in each arm, was determined for this research study based on sample size calculations for the outcome measure of medication understanding. Assuming an overall baseline rate of understanding of 65%, a sample of 300 patients per arm would enable the detection of an 11% absolute increase in medication understanding with 80% power. These differences were considered appropriate, based on the results of prior studies conducted by this study team [17,28,29]. This sample size also supports our exploratory analyses for the effects of the intervention on surrogate clinical outcomes. For example, a sample size of 600 provides adequate power (86%) to detect a difference of 3 mm Hg in mean systolic blood pressure by study arm, assuming a standard deviation of 12.

We will perform intent-to-treat analyses to determine the study results. Before conducting formal analyses, we will perform descriptive statistics to ensure adequate balance of patient characteristics across the two treatment arms (usual care and intervention) at baseline; *t* tests and *χ*^2^ tests will be used to evaluate differences across the two arms for continuous and binary variables, respectively. Generalized linear models will be used for data analyses using PROC GENMOD in SAS (version 9.2), specifying the logit link function for binary outcomes (medication understanding, reconciliation, adherence, and regimen consolidation) and the identity link function for continuous outcomes (blood pressure, hemoglobin A_1_c). The treatment group will be the independent variable of primary interest and will be modeled with usual care specified as the reference group. Any potential confounding covariates noted in the descriptive analysis will also be included in the models.

## Discussion

Innovative low-cost interventions are necessary to standardize and improve the currently fragmented system of patient medication information. If found effective, our strategy could serve as a model for providing language-appropriate, easy-to-understand medication information and support to patients to improve medication self-management in ambulatory care settings. With a focus on low health literacy, limited English proficiency, and multiple chronic conditions, our strategy seeks to promote safe and appropriate medication use among those at the greatest risk of poor medication-related outcomes. Our tools are designed to reduce medication discrepancies and patient misunderstanding by improving access to high-quality medication information and by prompting improved patient-provider communication. The strategy also involves producing an enhanced patient-centered medication summary in compliance with Joint Commission and Meaningful Use mandates [30,31]. Although the process of initially integrating the materials into the electronic health record is technical and time-intensive, once programming is completed, the intervention is low-cost, automated, and would require limited occasional adjustments compared with alternative approaches, particularly those involving additional staff support. As the majority of community health clinics now have an electronic health record, this strategy is increasingly feasible and can be disseminated to health centers across the nation [32].

The challenge of medication therapy management goes beyond effective risk communication by also seeking behavioral maintenance in support of sustained adherence to prescribed regimens. We hope to learn what our strategy can contribute to appropriate, safe medication use, how it might ultimately be disseminated, and also how it might be supplemented to address the multifaceted problem of medication self-management [33].

## Trial status

The study is currently enrolling participants. Enrollment began on 16 June 2013 and is anticipated to conclude in the summer of 2016.

## Abbreviations

CERT: Center for Education and Research on Therapeutics
PRN: pro re nata
